# Numerical simulation of stratospheric QBO impact on the planetary waves up to the thermosphere

**DOI:** 10.1038/s41598-022-26311-x

**Published:** 2022-12-15

**Authors:** A. V. Koval, N. M. Gavrilov, K. K. Kandieva, T. S. Ermakova, K. A. Didenko

**Affiliations:** 1 Saint-Petersburg State University, Saint-Petersburg, Russia; 2 Russian State Hydrometeorological University, Saint-Petersburg, Russia; 3 Institute for Meteorology, Leipzig University, Leipzig, Germany

**Keywords:** Atmospheric science, Atmospheric dynamics

## Abstract

With the help of numerical simulation, a detailed analysis of the dynamical effect of the stratospheric quasi-biennial oscillation (QBO) of the equatorial zonal wind on the planetary waves (PWs) up to thermospheric heights is carried out for the first time. The 3-dimensional nonlinear mechanistic model of middle and upper atmosphere (MUAM) is used, which is capable of simulating the general atmospheric circulation from the surface up to 300–400 km altitude. The amplitudes of stationary and westward travelling PWs with periods from 4 to 10 days are calculated based on ensembles of model simulations for conditions corresponding to the easterly and westerly QBO phases. Fluxes of wave activity and refractive indices of the atmosphere are calculated to analyze the detailed behavior of the PWs. The important result to emerge is that the stratospheric QBO causes statistically significant changes in the amplitudes of individual wave components up to 25% in the mesosphere-lower thermosphere and 10% changes above 200 km. This change in wave structures should be especially noticeable in the atmosphere during periods of low solar activity, when the direct contribution of solar activity fluctuations is minimized. Propagating from the troposphere to the upper atmosphere, PWs contribute to the propagation of the QBO signal not only from the equatorial region to extratropical latitudes, but also from the stratosphere to the thermosphere. The need for a detailed analysis of large-scale wave disturbances in the upper atmosphere and their relationship with the underlying layers is due, in particular, to their significant impact on satellite navigation and communication systems, which is caused by amplitude and phase fluctuations of the radio signal.

## Introduction

The quasi-biennial oscillation (QBO) of the equatorial zonal wind at stratospheric heights (e.g.,^1,2^) is one of the remarkable features of middle atmosphere dynamics. The QBO dominates the variability of the equatorial stratosphere at altitudes of ∼ 16–40 km, alternating easterly and westerly wind regimes with amplitude from − 30 to + 15 m/s and a period of about 2-years. The stratospheric QBO is driven primarily by equatorially trapped planetary waves forced by convective heating (e.g.,^3,4^) which propagate in opposite directions (eastward propagating Kelvin waves and westward propagating Rossby-gravity waves), and have nearly equal wave strengths^2^. The term QBO is usually referred not only to stratospheric variations of the zonal wind, but also to oscillations in solar activity (ionospheric QBO^5,6^). In this study, we focus on the stratospheric QBO, while the quasi-biennial oscillations in solar activity are not taken into account in the numerical model. In contrast to the analysis of observational data, numerical modeling allows us to isolate and study in detail the effect exerted by the stratospheric QBO. Although the QBO is a dynamical phenomenon occurring in the equatorial stratosphere, its influence also extends to the extratropical middle and upper atmosphere. For instance, the QBO impact at high latitudes is related to changes in the refractive index, restricting the propagation of Rossby waves to the lower latitudes, which leads to alterations in wave activity and the meridional mass circulation^7^. Recently, Koval et al.^8^ used numerical simulation to demonstrate the dynamical effects of the stratospheric QBO on the general atmospheric circulation up to the thermosphere. The observed changes in the meridional circulation, which is primarily driven by planetary waves (PWs), prompted us to perform a detailed analysis of the sensitivity of PW structures to the effects of the QBO. According to Koval et al.^9^, nonlinear interactions between PWs and global circulation during different QBO phases and corresponding changes in refractive indices and Eliassen-Palm (EP) fluxes may exert variations in PW amplitudes. The influence of the QBO on the stationary PW (SPW) structures calculated from the temperature fields from SABER/TIMED was discussed in^10^. It was shown that a quasi-biennial variation in SPW amplitudes is observed in the mesosphere, while the amplitude is greater during the easterly QBO (eQBO) compared with the westerly one (wQBO). This fact leaves an imprint on the formation of sudden stratospheric warmings (SSW). In particular, this could be the reason for the more frequent occurrence of SSWs during the eQBO^11^.

Despite a number of studies suggesting that SPWs cannot propagate deep to thermospheric heights (e.g.,^12–14^) due to the presence of critical layers and strong molecular dissipation, signatures of these waves are registered in all hydrodynamic fields (horizontal wind components, temperature, geopotential, total electron content) up to thermospheric/ionospheric heights. These waves do not propagate directly through the mesosphere—lower thermosphere (MLT) region due to before mentioned critical layers, but can be regenerated by nonlinear interaction with atmospheric tides^14^. These secondary SPWs in the thermosphere may also be influenced by the stratospheric QBO.

To date, a number of studies can be found in the scientific literature focused on the influence of the stratospheric QBO on the dynamics of the thermosphere/ionosphere. In particular, QBO signals in the f_o_F_2_ frequency oscillations were observed by Echer^15^, who registered clearly pronounced quasi-biennial oscillations in the ionosphere. However, it remained unclear whether these signals originate from the stratospheric QBO or from similar oscillations of solar activity. Using the TIE-GCM model and TIMED/SABER data, the investigation of biennial oscillations in ionospheric parameters was performed by Wang et al.^6^. They observed TEC, f_o_F_2_ frequency and migrating tides in the lower thermosphere. It was confirmed that these variations in the ionosphere are caused by both the ionospheric and the stratospheric QBO. At the same time, under the high solar activity conditions, the factor of ionospheric QBO is much stronger than the factor of stratospheric QBO.

In the present study, the numerical nonlinear mechanistic model MUAM is used, which can simulate atmospheric circulation including tides, SPWs, as well as westward and eastward propagating PWs from the surface up to 300–400 km altitude. This allows us to study the impact of the stratospheric QBO on the PWs in the thermosphere: the observed changes in structures of PWs at different QBO phases are transmitted to high altitudes, affecting the atmospheric circulation. At present, there are various methods and approaches for determining the QBO phases (see reviews in^2,8^, and references therein). The detection of zonal wind altitude, which choice affects the final research results, varies from 10 to 70 hPa in different studies^16^. In this study, we consider the vertical evolution of the equatorial zonal wind by applying the decomposition on empirical orthogonal functions (EOF). This approach is described in details in ^8^ when studying the QBO effect on atmospheric general circulation up to the lower thermosphere. A similar method was previously used in^17–19^, etc.

The quasi-biennial oscillations of the solar activity are not taken into account in the MUAM. This allows us to study the purely dynamical effects of the stratospheric QBO at altitudes up to the thermosphere to assess the contribution of the stratospheric QBO to wave disturbances in the thermosphere. Using this approach, we have previously demonstrated indirectly (based on the changes in wave-induced eddy meridional circulation) the propagation of the QBO influence to polar latitudes and to the thermosphere due to PW influence^8^.

The paper is structured as follows: Section “Introduction” gives a short review on the current state of the art concerning QBO research. Section “Methods and approaches” includes brief descriptions of the MUAM model and approaches used to take into account the QBO phases, as well as to analyze PW structures. Section “The simulated atmospheric general circulation” describes the general atmospheric circulation simulated with the MUAM. Section “Planetary waves amplitudes up to the thermospheric heights” is dedicated to the research of QBO-induced PW changes up to the thermosphere. Concluding remarks and discussion are presented in the last section.

### Methods and approaches

To study the changes in PWs up to the thermospheric heights under different QBO phases, a numerical simulation of the general atmospheric circulation from the Earth's surface to thermospheric heights is performed using a 3-dimensional nonlinear mechanistic model of the circulation of the middle and upper atmosphere (MUAM^20,21^). This model is based on the global circulation model COMMA, developed at the University of Cologne, Germany^22^, and uses a standard system of primitive equations in a spherical coordinate system (e.g.,^23^). The horizontal resolution of the model is 5° latitude and 5.625° longitude. A log-isobaric vertical coordinate *z* = − H*ln(p/p_*0*_*)*, where p_0_ is the surface pressure and *H* is the pressure scale height, is used. The important advantage of MUAM, the mechanistic model with a relatively low resolution, is its ability to perform rapid simulations of dynamic processes up to heights of 300–400 km without taking significant processor time. Moreover, MUAM allows to reproduce global resonance properties of the atmosphere^20^, which is important for an adequate modeling of stratospheric vacillation cycles (see also^24^) responsible for formation of global-scale atmospheric nonlinear wave interactions.

The MUAM model is capable of reproducing SPWs, atmospheric normal modes (NMs)^25^, and atmospheric migrating and non-migrating tides^26^. SPW amplitudes at the lower boundary are calculated from the geopotential heights in the lower atmosphere obtained from the UK Met Office stratospheric assimilation data^27^. As far as MUAM has relatively low spatial resolution and does not reproduce tropospheric weather, tropospheric sources of westward travelling atmospheric NMs are set with additional terms in the heat balance equation, having a form of time-dependent sinusoidal components with zonal wavenumbers *m* = 1 or *m* = 2. For setting the latitude structures of NM components, the Hough functions are used, obtained with the method described by Swarztrauber and Kasahara^28^. The periods of the NMs correspond to the resonance periods of atmospheric response to the wave forcing at the lower boundary^29^. In the current model experiments, NMs are defined as (1,1), (1,2), (1,3), (2,1) and (2,2) (we use classification proposed by Longuet-Higgins^30^), which have periods of about 5, 10, 16, 4 and 7 days, respectively. The NM sources used in the MUAM provide amplitudes of the simulated NM that are comparable to the observed ones in the stratosphere^31^. The MUAM includes a parameterization of thermal and dynamical effects produced by mountain waves^32^ and a spectrum of non-orographic gravity waves^33^. Numerous analyses have been carried out to validate MUAM, which have shown that it correctly reproduces the structure of the atmospheric circulation, PWs, and tides (e.g.,^8,9,21,34,35^), sudden stratospheric warmings^36^, as well as the resonance properties of the atmosphere (normal atmospheric modes)^20^.

To assess the statistical significance of the results of simulations, two 16-member ensembles of the MUAM model calculations are obtained for conditions corresponding to the eQBO and wQBO. The ensembles are formed from MUAM runs corresponding to different phases of vacillations between the mean wind and PW in the middle atmosphere. These phases in MUAM are controlled by changes in the onset date of diurnal variations in solar heating and generation of atmospheric NMs^20^. The initial and background conditions for all model runs are assumed to be identical. Monthly-mean PW amplitudes, mean flow intensity, and winter stratospheric temperature can change significantly from one model “run” to another. This variability in model simulations is interpreted as interannual^20,21^. Physical and hydrodynamic processes taken into account in the current version of the MUAM and the scheme of a numerical experiment are described in^8^.

### Accounting of QBO in MUAM

To reproduce the stratospheric QBO, relaxation (nudging) of the modeled zonally averaged zonal wind fields to observations is used in the MUAM. Therefore, background and initial hydrodynamic fields for the years corresponding to the different QBO phases must be specified for the simulations. To prepare the initial and background conditions for the numerical simulations, we use QBO phase determination based on the decomposition of observed equatorial zonal wind variations with empirical orthogonal functions (EOF). This approach allows us to analyze the vertical evolution of the equatorial zonal wind profile. This decomposition is basically similar to Fourier analysis, however, instead of using sinusoids, EOF involves basis functions determined from the analyzed fields. In general, this allows to detect large-scale spatio-temporal patterns that are not necessarily represented by waves. For the study of the QBO signal in the zonal wind field, data of the Japanese 55-year reanalysis (JRA-55^37^) was used. The anomalies of the zonal wind are considered relative to the values averaged over 1958–2019. Using this procedure (which is described in^8^), we selected two sets of years related to typical westerly (1983, 1985, 1993, 1995, 1997, 1999, 2002, 2004, 2008, 2013) and easterly (1987, 1989, 1996, 1998, 2000, 2003, 2005, 2007, 2010, 2012) QBO phases and calculated average zonal-mean distributions of zonal wind and temperature for both QBO phases. During the simulation experiments, the additional terms in the momentum equation for zonal wind velocity are used in the MUAM, proportional to differences between calculated and observed zonal mean winds at latitudes from 17.5°S to 17.5°N and at altitudes from 0 to 60 km. The proportionality constant is a value related to the characteristic relaxation time (~ 5 days) of the calculated hydrodynamic fields with respect to the observed ones. Simulations of the general atmospheric circulation for January–February were carried out with the specified QBO phases using the method described above.

### Eliassen-Palm flux

The structure of the mean flow can alter the propagation of PWs and tides, while these waves produce changes in momentum of the mean flow. Calculation of the EP Flux, which represents zonal mean flow–wave interaction, allows one to diagnose the sources and sinks of momentum during the easterly and westerly QBO phases^4^. To quantify the changes in circulation and assess possible PW contributions to its structure, the meridional and vertical components of the Eliassen-Palm flux (EP flux) ${F}_{m}=({F}_{m}^{(\varphi )},{F}_{m}^{(z)})$ were calculated according to the formulas^4^:

1 $${F}_{m}^{(\varphi )}=\mathit{cos}\varphi ({\bar{u}}_{z}\frac{\overline{{v}^{^{\prime}}{\theta }^{^{\prime}}}}{{\bar{\theta }}_{z}}-\overline{u{^{\prime}}v {^{\prime}}})$$

2 $${F}_{m}^{(z)}=\mathit{cos}\varphi \left(\left(f-\frac{(\bar{u}\mathit{cos}\varphi {)}_{\varphi }}{a\mathit{cos}\varphi }\right)\frac{\overline{v{^{\prime}}\theta {^{\prime}}}}{{\bar{\theta }}_{z}}-\overline{{w}^{^{\prime}}{u}^{^{\prime}}}\right)$$

Here, overbars and primes denote zonal-mean values and deviations from the zonal-mean values, respectively; lower indices mark partial derivatives; *u*, *v* and *w* are zonal, meridional and vertical wind components; $\theta =Texp(\frac{g}{Cp}{\int }_{0}^{h}\frac{\mu }{T}dh)$ is potential temperature; *h* is the geopotential height; *C*_*p*_ is the heat capacity at constant pressure; *φ* is latitude; *f* is the Coriolis parameter. The EP flux divergence was also calculated using the formula by^4^:

3 $$\nabla {F}_{m}=\frac{1}{a\mathit{cos}\varphi }\frac{\partial }{\partial \varphi }\left({F}_{m}^{\left(\varphi \right)}cos\varphi \right)+\frac{\partial {F}_{m}^{(z)}}{\partial z}$$

The divergence of the EP flux shows the net drag of the zonal-mean flow by PWs. For example, negative values of the EP flux divergence (i.e., convergence) correspond to a westward drag on the mean wind.

### Refractive index

Another important PW characteristic we use to broaden the analysis of wave interactions in the atmosphere, is quasi-geostrophic zonal-mean refractivity index squared, *n*^*2*^. It was first introduced by Matsuno^38^, who found that PWs propagate along the waveguides: areas of large positive values of this index. Refractive index is calculated by the formulae (e.g.,^39^):

4 $${n}^{2}(\varphi ,z)=\frac{\overline{{q }_{\varphi }}}{\mathrm{u}-\mathrm{c}}-{\left(\frac{m}{a\mathit{cos}\varphi }\right)}^{2}-{\left(\frac{f}{2NH}\right)}^{2},$$

where $\overline{{q }_{\varphi }}$ is the latitudinal gradient of zonal-mean potential vorticity; $c=2\pi a\mathrm{cos}\phi /m\tau$ is the zonal phase velocity of PW mode; *τ* is the wave period; *a* is the Earth’s radius, *N* is the Brunt-Vaisala frequency, and *H* is the atmospheric pressure scale height. For the zonal-mean potential vorticity ${\bar{q}}_{\varphi }$ gradient shown in Eq. (4), we use formulae from^39^:

5 $$\stackrel{-}{{\bar{q}}_{\varphi }}=\frac{2\Omega \mathit{cos}\varphi }{a}-\frac{1}{{a}^{2}}{\left(\frac{{\left(\bar{u}\mathit{cos}\varphi \right)}_{\varphi }}{\mathit{cos}\varphi }\right)}_{\varphi }-\frac{{f}^{2}}{\rho }{\left(\rho \frac{{\bar{u}}_{z}}{{N}^{2}}\right)}_{z},$$

where $\rho ={\rho }_{0}\mathrm{exp}(-z/H)$ is the standard density in log-pressure coordinates, *ρ*_*0*_ is the sea-level reference density.

## The simulated atmospheric general circulation

**Figure 1:**
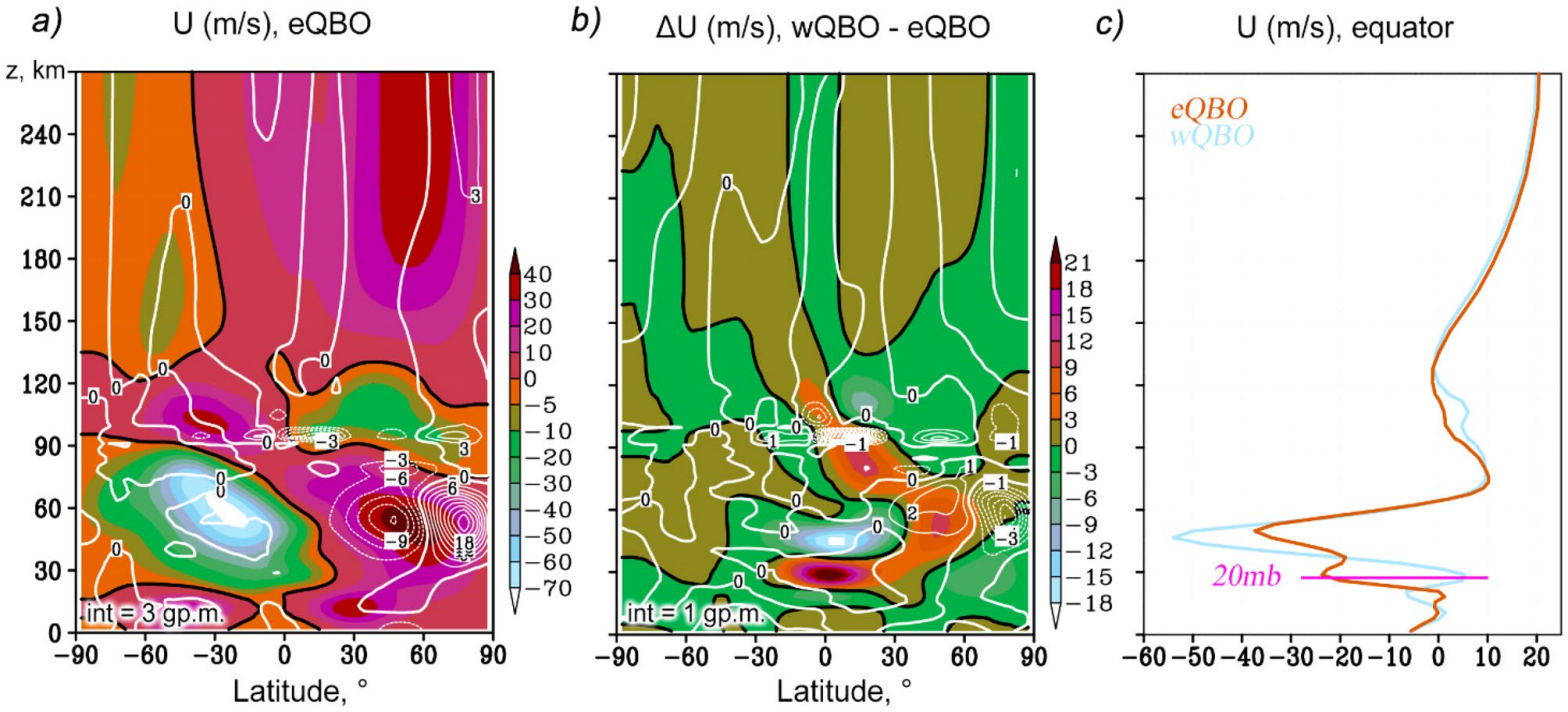
Latitude-height distributions (shaded) of the zonal-mean wind (m/s, **a**), according to MUAM ensemble for January–February, eQBO; respective zonal mean wind increments due to change from wQBO to eQBO (**b**); vertical profiles of the equatorial zonal wind for both QBO phases (**c**). Contours in the panels (**a**, **b**) reveal EP-flux divergence and its increments in 10^–2^ m^2^/s^2^/day, respectively.

Figure 1a,b show the latitude-height distributions of the zonal mean zonal wind according to the MUAM results for January–February under the eQBO conditions, and its increment (wQBO–eQBO). The contours in Fig. 1a and b reveal EP-flux divergence and its increments, respectively, calculated according to the formula (3). A positive (eastward) acceleration in the zonal mean flow corresponds to a divergence of the wave activity and thus represents a source of the wave momentum, while a deceleration in the zonal mean flow corresponds to a convergence of wave activity and therefore to its sink towards the mean flow. These processes are observed primarily in the northern strato-mesosphere and at the MLT heights (Fig. 1a). In the mid-latitude winter stratosphere, the wave action contributes to the deceleration of the eastward wind, while at high latitudes its acceleration is observed. The structure of jet streams in the middle atmosphere is also influenced by other factors, such as the inhomogeneity of the heating of the atmosphere, which leads to the formation of meridional temperature gradients. If we consider the distributions of the zonal wind increments and EP flux divergence due to changes in the QBO phase in Fig. 1b, the acceleration of the stratospheric zonal wind in the latitude range of 30°–60°N under wQBO is explained by enhancement in the EP flux divergence and also by a decrease in temperature (not shown), which contributes to a weakening in the meridional temperature gradient. At higher latitudes, on the contrary, a deceleration of the zonal flow is observed, corresponding to a decrease in the EP flux divergence and an increase in the meridional thermal gradient.

Figure 1c shows profiles of the zonal mean zonal wind averaged over the equatorial region (± 2.5°) for the both QBO phases. As was discussed by Koval et al.^8^, the QBO phase is determined by the direction of the main component of the EOF expansion of the equatorial zonal wind field at a level of 20 mb (indicated by the horizontal pink line in Fig. 1c): it is clearly seen that the zonal wind at this level is directed eastward and westward for the wQBO and eQBO, respectively. There are also substantial differences in the speed of the zonal wind in the altitude range of 40–60 km, and in the MLT region, which are primarily caused by wave action (see below).

A statistical significance of the obtained increments in Fig. 1b was calculated based on the paired Student’s t-test applied for 32 values (16 pairs of model runs × 2 months). The calculation showed 95% statistical significance of nonzero differences for values of |∆U| > 2 m/s. This is true for most regions in the Fig. 1b, except for the intervals just along zero contours. In the study by Koval et al.^8^, the simulated with the MUAM circulation calculated in this way for both QBO phases was compared with the data of the MERRA-2 reanalysis^40^ for the corresponding set of years, as well as with empirical models of the horizontal wind HWM-14^41^ and temperature NRLMSIS 2.0^42^. A good correlation was noted between the simulated hydrodynamic fields and the specified data sets.

## Planetary waves amplitudes up to the thermospheric heights

The SPW and NM amplitudes and phases were calculated based on the simulated geopotential height fields with the MUAM for each of the 32 model “runs” (two 16-member ensembles for eQBO and wQBO, respectively). We use two-month period (January–February), dividing it into four 15-day subintervals. For each subinterval, the longitude-time Fourier transform and the least squares fitting are applied to calculate amplitudes and phases of the SPWs and NMs. To perform statistical analysis, we received 64 pairs of SPW amplitudes with zonal wavenumbers *m* = 1 and *m* = 2 (SPW1 and SPW2 hereafter) and NM amplitudes with periods approximately *5* and *10* days (*m* = 1), also *4* and *7* days (*m* = 2).

**Figure 2:**
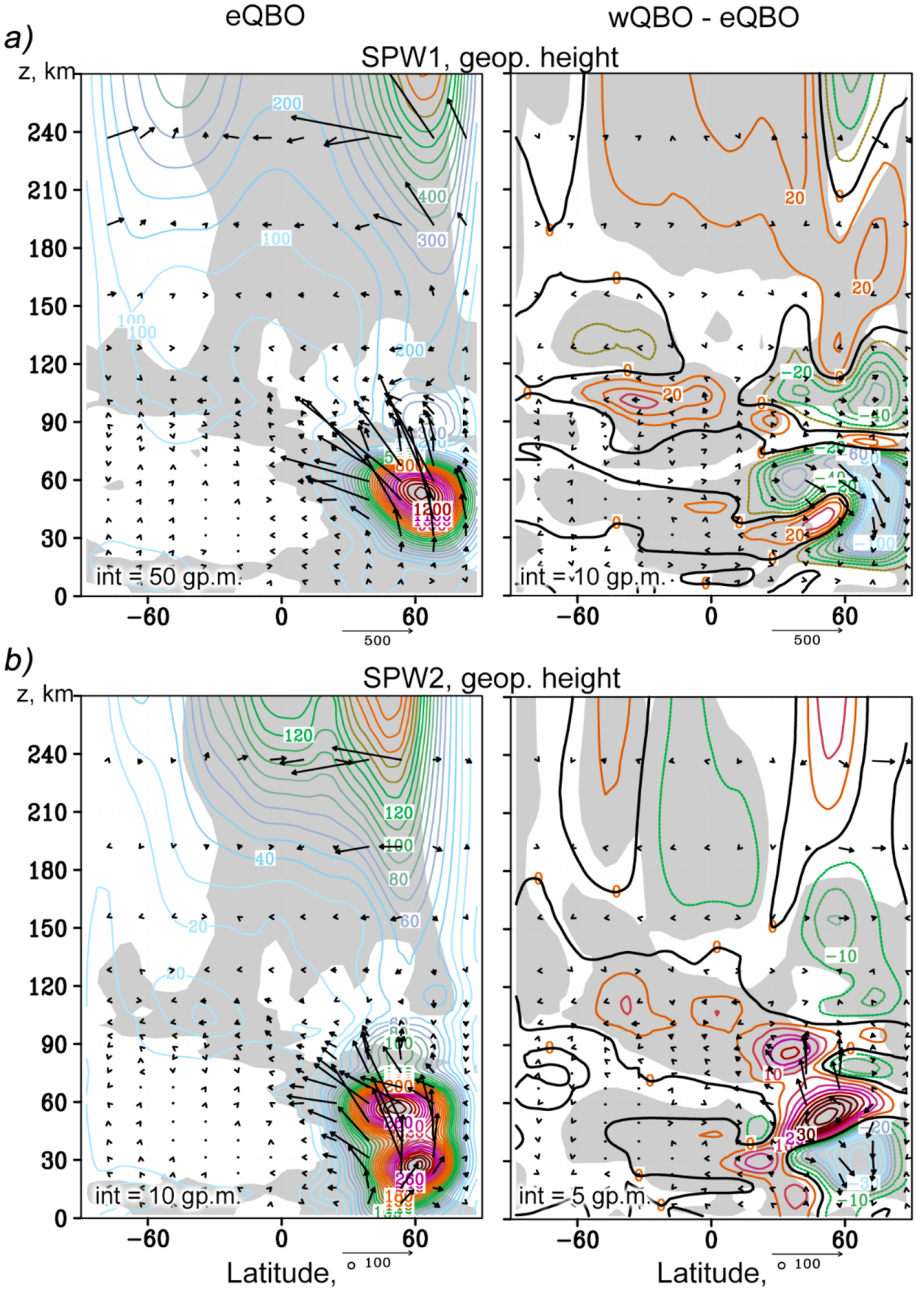
Amplitudes of the geopotential height variations caused by SPW1 and SPW2 in gp. m. (**a** and** b**, left, respectively) for the MUAM ensemble for January–February, eQBO; respective amplitude increments due to change from wQBO to eQBO (right). Arrows show EP flux (left) and its increments (right) in e^z/70^ m^2^/s^2^ (vertical component is multiplied by 200). Gray background shows waveguide (left) and 95% statistical significance (right). Intervals between contours are indicated in the left bottom corner of each plot.

The left panels of Fig. 2 show the amplitudes of geopotential height variations by means of SPW1 and SPW2, calculated using the MUAM results for the eQBO. The arrows represent vertical and meridional components of the EP flux, and the gray background highlights the waveguides: regions with positive refractive indices *n*^*2*^. PWs should propagate in atmospheric regions where *n*^*2*^ > 0 and weaken in regions, where *n*^*2*^ < 0 (e.g.^4^). Therefore, PW waveguides are located inside the surfaces where *n*^*2*^ changes its sign. These boundaries are located usually near PW critical levels, where $\overline{u }=c$ and ${n}^{2} \to \infty$ according to Eq. (4).

**Figure 3:**
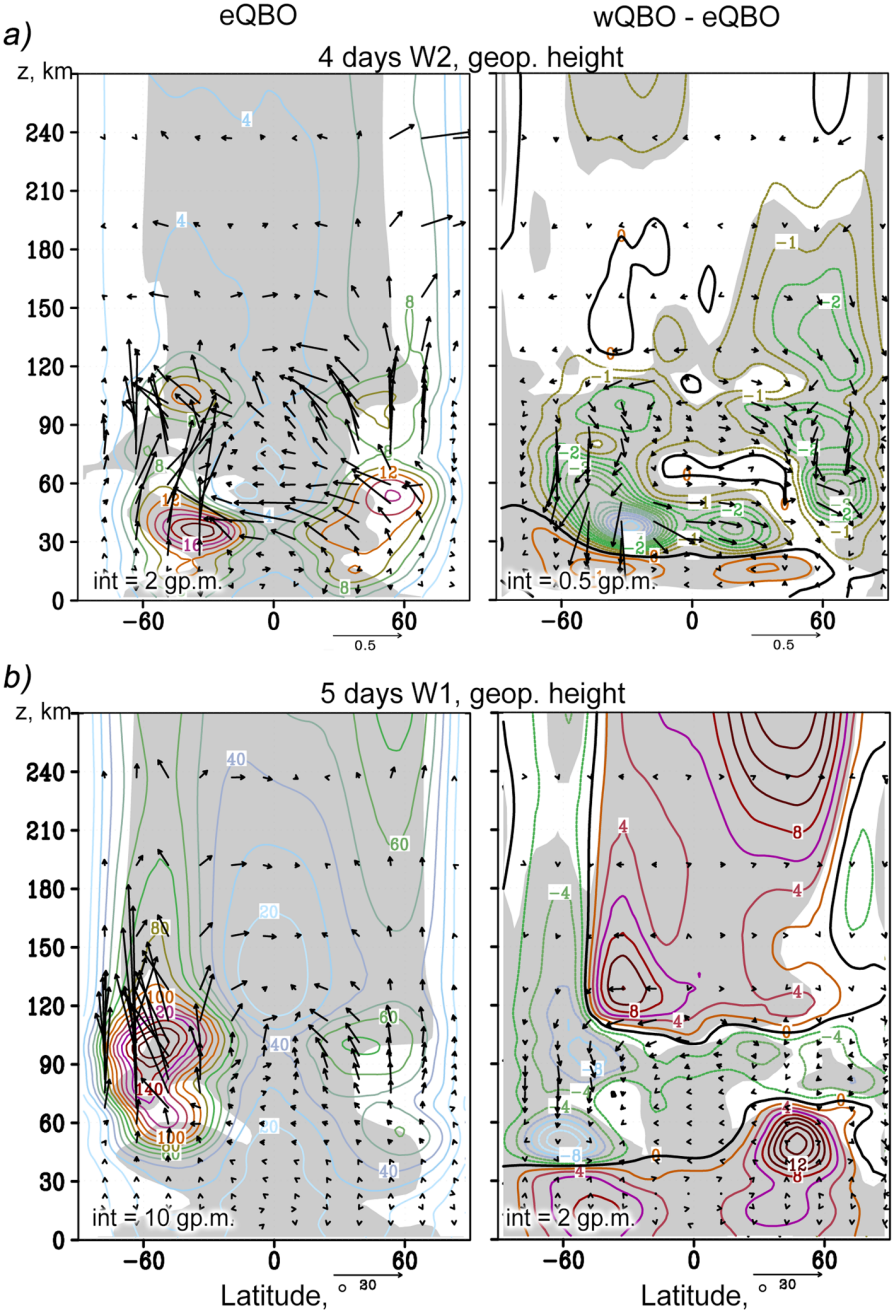
The same as Fig. 2 but for the westward travelling atmospheric NMs: *τ* = 4 days,* m* = 2 (**a**); *τ* = 5 days*, m* = 1 (**b**).

**Figure 4:**
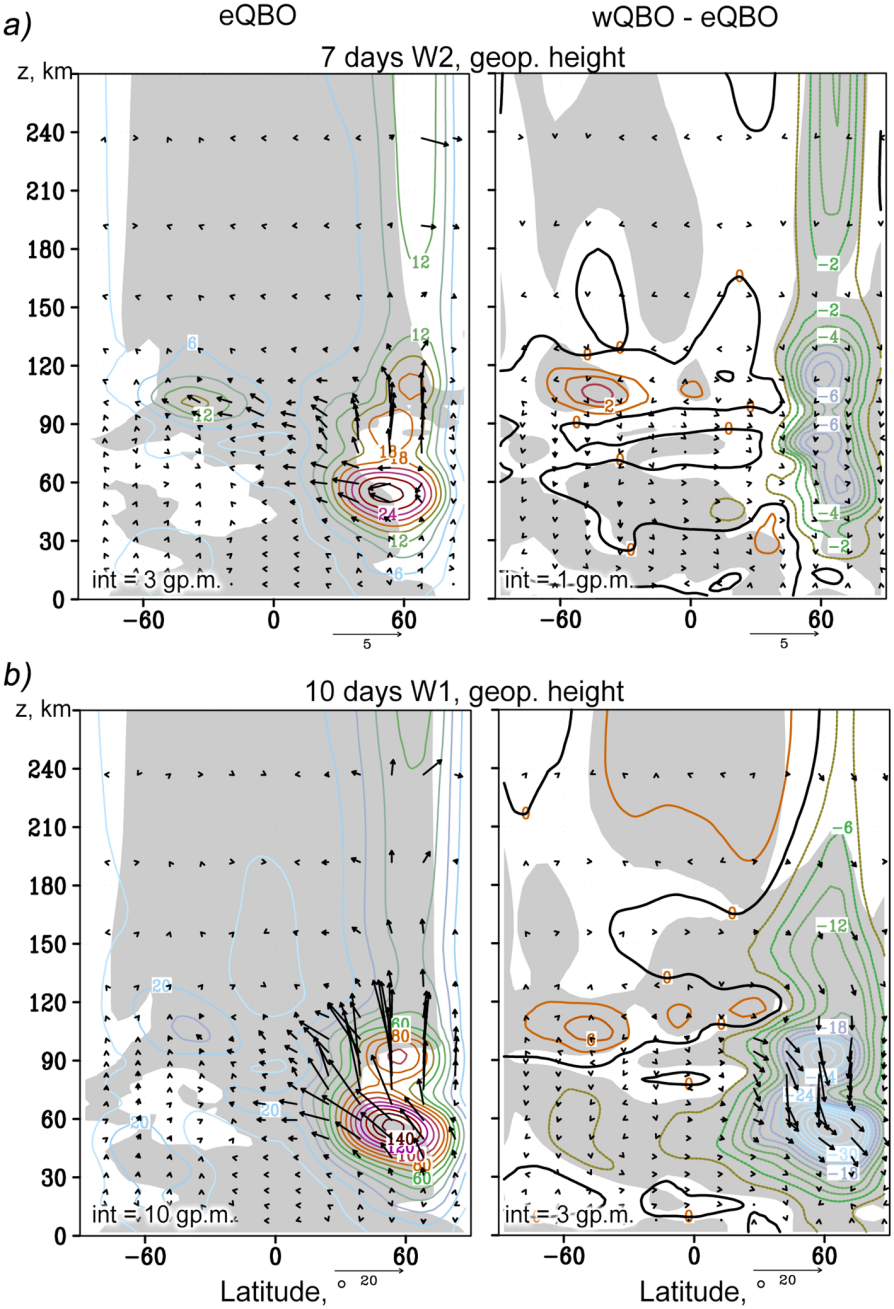
The same as Fig. 2 but for the westward travelling atmospheric NMs: *τ* = 7 days,* m* = 2 (**a**); *τ* = 10 days, *m* = 1 (**b**).

It can be seen that SPWs propagate upward from the troposphere, where they are generated, along the waveguides, through the circulation structures characteristic of the winter season (Northern Hemisphere). In the upper stratosphere and mesosphere, the waveguides, and hence the SPW, cross the equator and propagate upward in both hemispheres. EP flux represents the wave activity flux and indicates the predominant wave propagation in the meridional plane. In the stratosphere of the Northern Hemisphere the SPW amplitudes are maximum and are accompanied by maximum EP fluxes that are directed upward and toward the equator. The simulated SPW amplitudes at altitudes up to 60 km were compared with those calculated using the reanalysis data of meteorological information MERRA-2 and UK Met Office for the same sets of years that were used for the nudging in MUAM. A good correlation was found. The general structure of SPWs (Fig. 2) and NMs (Figs. 3 and 4) also corresponds to satellite measurements, which was discussed in^35^. Above 90 km, the waveguides in the Northern Hemisphere are interrupted due to a change in the direction of the zonal wind, and the EP fluxes sharply decrease. Above this layer, the SPW is much weaker due to enhancement of kinematic viscosity and thermal conductivity, which finally lead to barriers in their direct propagation. SPWs cannot propagate in the thermosphere directly (e.g.,^12–14^), but only through secondary excitation. In the thermosphere, the SPW structure is more uniform in latitudes, and the EP fluxes are directed predominantly upward and from the poles to the equator. Starting from the lower thermosphere, the SPW amplitudes increase with height. In this case, SPW1 is maximum at the middle latitudes of both hemispheres, while SPW2 propagates mainly in the equatorial region and in the Northern Hemisphere. SPW2 waveguide in the thermosphere is substantially narrower than SPW1 waveguide.

The right panels of Fig. 2 reveal the increments of the corresponding SPW amplitudes and EP fluxes due to a change in the QBO phase. The areas corresponding to 95% of the statistical significance of non-zero amplitude increments are marked with a gray background. Statistical significance was calculated using paired Student's t-test, based on 64 pairs of values (16 model runs × 4 time subintervals). In the right side of Fig. 2a, there is a statistically significant weakening of the SPW1 in the extratropical northern (winter) stratosphere. It is accompanied by a weakening of the EP flux in this area. In the right side of Fig. 2b, the weakening of the SPW2 wave activity in the lower northern stratosphere is accompanied by its intensification above 40 km, which leads, respectively, to a decrease and an increase in the SPW2 amplitude. Significant changes in the amplitudes of SPW1 and SPW2 are also observed in the altitude range of 80–120 km in both hemispheres. The SPW amplitudes increase during wQBO in the Southern Hemisphere, while in the Northern Hemisphere they generally decrease, and the increments can reach 25%. At the altitudes above 200 km, statistically significant changes in the SPW amplitudes caused by the impact of the stratospheric QBO are also observed. At middle and low latitudes, an increase in SPW1 occurs at wQBO, which can exceed 20%, and at high northern latitudes, the amplitudes decrease, which is accompanied by corresponding changes in the EP fluxes.

Figures 3 and 4 show distributions similar to Fig. 2, but for westward propagating NMs having following characteristics: τ = 4 days (*m* = 2) and τ = 5 days (*m* = 1) in Fig. 3 and τ = 7 days (*m* = 2) and τ = 10 days (*m* = 1) in Fig. 4. Their waveguides in the middle atmosphere are much wider than those of SPWs, because structure of waveguides, according to Eq. (4) is determined by their non-zero (specifically, negative) phase velocities. This makes it possible for waves to propagate in the Southern Hemisphere, in the area of westward zonal wind, provided that this wind is weaker than the PW phase velocity. Garcia et al.^43^ analyzed the 5-day PW in the mesosphere and lower thermosphere observed with the SABER/TIMED in years 2001–2004. They found large temperature amplitudes of the 5-day PW up to 2.5–3.5 K both in the Northern and Southern Hemispheres. The amplitudes are comparable with those shown in left Fig. 3b and confirm propagation of westward 5-day NM in both hemispheres.

The 4- and 5-day waves shown in the left Fig. 3a and b, respectively, have large phase velocities, which allows them to have significant amplitudes in the summer stratosphere, in contrast to the slower 7- and 10-day waves shown in Fig. 4a and b. Remarkable feature of the 4-day wave is its strong horizontal transfer of wave activity from the winter to the summer hemisphere (see left Fig. 3a), i.e. this wave should make a significant contribution to the interhemispheric coupling at stratospheric heights. In the thermosphere, above about 120 km, the amplitudes of all PWs decrease. Atmospheric NMs, like the SPWs, cannot propagate upward directly. After the secondary excitation, in the thermosphere PWs are observed with the similar periods and the same wavenumbers as NMs in the middle atmosphere. At high altitudes, PWs propagate in the middle northern latitudes, except for the 5-day wave, whose amplitudes are comparable in the mid-latitude regions of both hemispheres.

In the right panels of Figs. 3 and 4, the increments in the amplitudes of the NM and EP fluxes due to a change in the phase of the QBO are shown. In the stratosphere, the changes in the NM structure generally correspond to earlier studies (e.g.,^44^). In particular, during wQBO, an increase in the amplitudes of the 4-day wave (Fig. 3a) and the 5-day wave (Fig. 3b) is observed in the lower stratosphere of both hemispheres, and a decrease in the upper stratosphere and mesosphere. These changes are accompanied by corresponding amplifications and attenuations of the vertical component of the EP flux. In the thermosphere, the 5-day wave under the wQBO intensifies, except for the high-latitude belt in the Southern Hemisphere, where the opposite effect is observed.

The structure of lower-frequency 10- and 7-day waves presented in Fig. 4 is significantly different from the 5- and 4-day ones discussed above. First, their amplitudes in the stratosphere are maximum in the Northern (winter) Hemisphere. The strongest vertical EP fluxes are directed along the northern boundary of the waveguides. In addition, a strong flux of wave activity is observed from the lower stratosphere upward and to the South, crossing the equator. Second, the change in the QBO phases has a similar effect on the 7- and 10-day waves, which is expressed primarily in their weakening during the wQBO in the Northern Hemisphere, in the entire altitude range up to 270 km. At the same time, on the contrary, an increase in these wave components is observed at the heights of the MLT region (90–130 km).

## Discussion

In addition to the structure of the atmospheric circulation itself, which is expressed in changes in the EP fluxes under different QBO phases, an increase in the background temperature during the eQBO, discussed in^8^, can also have an effect on the PW amplitudes in the thermosphere. This increase is caused, first of all, by the slowdown in the meridional transfer of air masses from the warmer summer thermosphere during the westerly QBO. This warming causes an increase in the vertical temperature gradient and contributes to an increase in the Brunt-Vaisala frequency *N*. The wave activity flux, ***F***_***a***_, can be represented in the form of ***F***_*a*_ = ***c***_*g*_A, where ***c***_*g*_ is the group velocity and *A* is the wave activity density^4^. The vertical component of group velocity *c*_gz_ ~ *k*^−1^, where *k* is the vertical wavenumber. An increase in *N* leads to increasing *k,* according to the PW dispersion relation. Discontinuity of the vertical component of the wave activity flux *F*_*az*_ at increasing *k* requires respective increase in the wave activity *A* and in PW amplitudes^9^.

Consideration of the left panels in Figs. 2, 3, and 4 shows that most simulated maxima of PW amplitude in the middle atmosphere and thermosphere are located inside and at the edges of grey-shaded regions of waveguides spanning from the lower to the upper atmosphere. As described in “Planetary waves amplitudes up to the thermospheric heights” section, these waveguides correspond to regions of positive refractive index *n*^2^ > 0 produced by zonal mean fields of wind and temperature, where PW energy can propagate vertically (e.g.,^3,4^). Strong eastward winds may produce interruptions of the PW waveguides in the mesosphere and lower thermosphere, which is more noticeable in the left panels of Fig. 2 for stationary waves: SPW1 and SPW2 at altitudes 60–140 km in the entire winter Northern Hemisphere. Respective interruptions of waveguides for westward propagating NMs in the left panels of Figs. 3 and 4 exist in the Northern Hemisphere at latitudes higher than 40–50° N. On the other hand, in the left panels of Figs. 2 and 4 one can see amplitude maxima of all considered PW modes in the northern thermosphere above the waveguide interruptions. This raise questions how PW energy can penetrate to the thermosphere above layers, where PW propagation is prohibited.

One way for wave energy penetration to the thermosphere could be PW propagation from the winter (northern) middle atmosphere to the summer (southern) thermosphere along waveguides, which cross the equator at altitudes 60–90 km according to the left panels of Figs. 2, 3, and 4. In the thermospheric waveguides, PW modes can propagate back to the high-latitude winter (northern) thermosphere at high altitudes.

Another possibility, which is first noted by Laštovicka et al.^14^ and then actively discussed in the literature (e.g.,^45^) is modulation of tides (and possibly gravity waves) by PW modes in the waveguide located in the winter middle atmosphere in the left panels of Figs. 2, 3, and 4. These PW-modulated tides and gravity waves can then propagate to the winter thermosphere and induce there so called “planetary wave-type oscillations” (PWTO), which are observed by different methods (e.g.,^46^).

**Figure 5:**
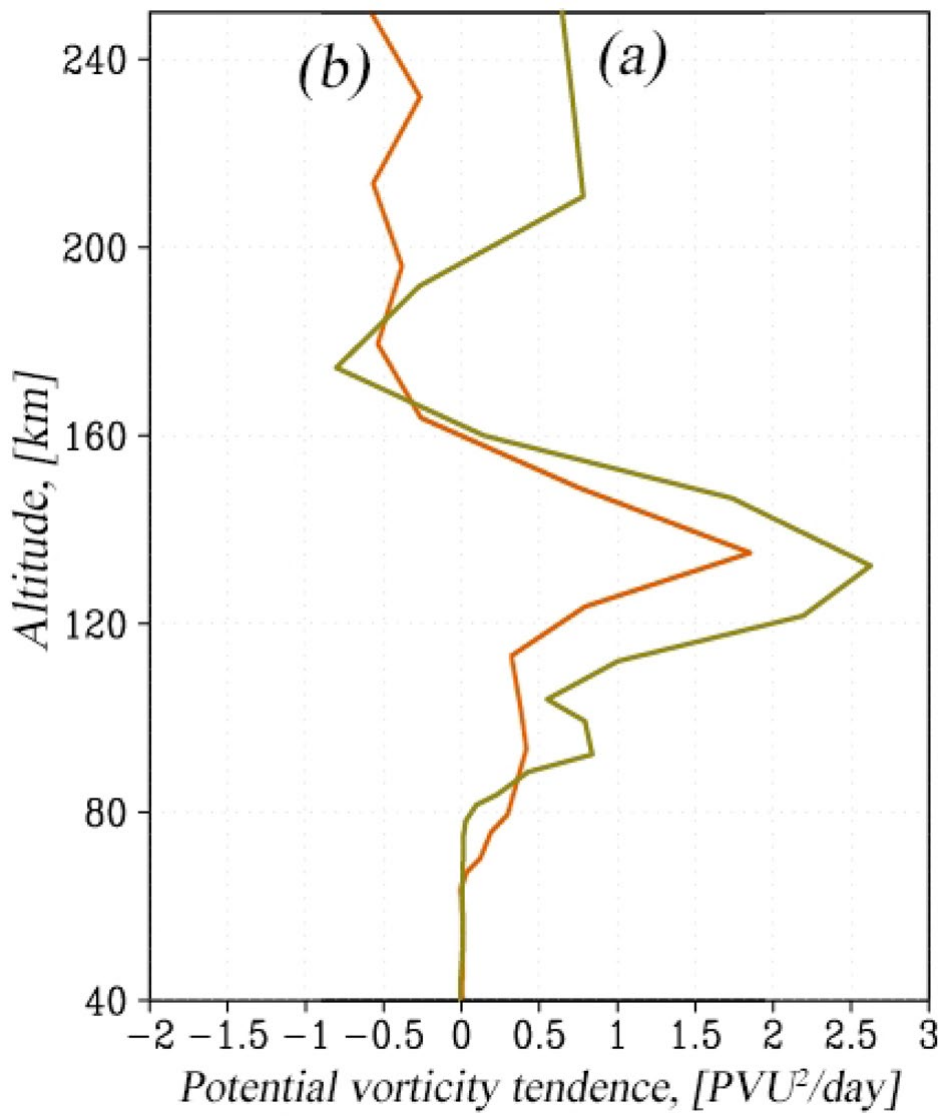
Vertical profile of the sum of all terms in the perturbed potential enstrophy balance equation for non-migrating diurnal tide (averaged over latitudes and longitudes, PVU^2^/day)—(a). Sum of terms describing nonlinear interaction between SPW1 and migrating diurnal tide—(b).

To diagnose this mechanism, we used the perturbed potential enstrophy (Ertel's potential vorticity squared) balance equation^47,48^. This equation includes terms describing the interaction of the wave with the mean flow, the advection of potential enstrophy and the divergence of its flow, also the terms describing the nonlinear interactions between the PWs. This equation is convenient from the point of view of estimating the relative contribution of various components to the generation of a certain wave mode. Such an approach has been applied to the study not only of nonlinear SPW interactions, but also of nonlinear interactions between migrating tides^49^. When two waves having frequencies *ω* and zonal numbers *m* interact, a new (secondary) wave arises, in which the frequency and wave number are the sum or difference of the corresponding values of the primary waves (e.g.,^50^). For example, during the interaction of SPW with *m* = 1 with a migrating diurnal tide (period of 24 h, *m* = 1), a non-migrating diurnal tide with *m* = 2 is formed. To demonstrate this mechanism, we calculated all terms in the perturbed potential enstrophy balance equation for non-migrating diurnal tide. The formulas for calculation are analogous to ones presented by Didenko et al.^49^. Green curve (a) in Fig. 5 shows vertical profile of the sum of all terms of this equation (averaged over latitudes and longitudes). Orange curve (b) shows sum of terms describing nonlinear interaction between SPW1 and migrating diurnal tide, i.e., describing potential vorticity transfer to the secondary wave. It can be seen that generation of the non-migrating tide begins at about 70 km, increases to the altitude of about 140 km and decreases at upper levels. In this case, this nonlinear contribution becomes negative at altitudes above 160 km, i.e., there is a reverse transfer of potential vorticity (towards the SPW1 and/or the migrating tide). This reverse process should contribute to the formation of tertiary SPW (or PWTO) due to the interaction of migrating and non-migrating tides. A more detailed analysis, including also semidiurnal migrating tide modulation, using the balance equation of the disturbed potential enstrophy will be the object of our further research, since it is outside the scope of this study.

## Conclusion

Using multiple MUAM model “runs”, numerical simulation of the atmospheric general circulation for January–February has been performed to calculate amplitudes of planetary waves and to investigate differences in their structures caused by stratospheric QBO up to thermospheric heights. The series of carried out experiments allowed us to study the dynamic influence of the stratospheric QBO on wave structures up to high altitudes, excluding other factors, such as, for example, biennial oscillations in solar activity. Such study enhances understanding of the nature of atmospheric processes, including the vertical interaction between the middle and upper atmosphere. The easterly and westerly QBO phases were determined based on decomposition of equatorial zonal wind profile with empirical orthogonal functions. This technique allows considering not only the change in the direction of the zonal wind at a certain altitude, but also time evolution of the vertical structure of the zonal wind in the entire stratosphere.

The results of the studies showed that the stratospheric QBO, which is localized in the equatorial region, can not only have a significant effect on the amplitudes of SPWs and NMs in the extratropical stratosphere (which confirms current knowledge), but also cause statistically significant changes in the amplitudes of these waves in the upper atmosphere. In particular, substantial changes in the amplitudes of planetary waves (up to 25%) were revealed at altitudes of the MLT region. Above 200 km, changes in the amplitudes of individual wave components can exceed 10%. These changes in the wave structures should be especially noticeable in the atmosphere at a low level of solar activity, when the direct contribution of solar activity fluctuations (e.g., ionospheric QBO) is minimized.

The main contribution to the change in the PW structure is made by a change in the atmospheric circulation, which manifests itself in variations in the EP flux under different QBO phases, as well as changes in the vertical temperature profiles caused by a change in the meridional circulation in the thermosphere.

In order to achieve further progress in the understanding of atmospheric motions and the vertical coupling between the atmospheric layers, we plan to consider the dynamical effect of the stratospheric QBO on atmospheric migrating and non-migrating tides, as well as the "fast" PWs (high-frequency waves with periods of up to 5 days and zonal wavenumbers 1–3) penetrating directly from the lower atmosphere to the thermosphere and affecting the composition and dynamics of the ionosphere.Another important direction is diagnostics of the possibility of the PW-modulation-tide mechanism in the MUAM numerical model, with the help of the perturbed potential enstrophy balance equation.

## Data Availability

All data presented in the paper can be freely accessed at https://doi.org/10.5281/zenodo.5596370. According to the statement 1296 of the Civil Code of the Russian Federation, all rights on the MUAM code belong to the Russian State Hydrometeorological University (RSHU). To get access to the computer codes and for their usage a reader should get a permission from the RSHU Rector at the address 79, Voronezhskaya street, St. Petersburg, Russia, 192007, phone: 007 (812) 372–50-92. The authors will assist in getting such permission.
